# Chronic pyloric obstruction caused by Flammulina mushroom: A rare case report

**DOI:** 10.1097/MD.0000000000040637

**Published:** 2024-11-29

**Authors:** Zhaohui Liu, Ruinuan Wu, Zepeng Wang, Dayong Sun, Xiangyu Wang

**Affiliations:** a Department of Gastroenterology, The First Affiliated Hospital of Shenzhen University, Shenzhen Second People’s Hospital, Shenzhen, China; b Department of Pathology, The First Affiliated Hospital of Shenzhen University, Shenzhen Second People’s Hospital, Shenzhen, China; c Marshall Laboratory of Biomedical Engineering, Shenzhen University, Shenzhen, China.

**Keywords:** endoscopy, Flammulina mushroom, pyloric obstruction

## Abstract

**Rationale:**

We report a rare case of chronic pyloric obstruction lasting 4 years. During the endoscopy examination, the cause was found to be foreign matter blocking the pylorus. We assessed the patient’s medical history and ultimately considered chronic pyloric obstruction caused by blockage of the Flammulina mushroom.

**Patient concerns:**

A man in his early 30 presented to the emergency department of the hospital due to recurrent vomiting for 4 years that had aggravated for 2 days.

**Diagnoses:**

Emergency CT indicated enlargement of the gastric cavity, numerous gastric contents in the gastric cavity, and suspicion of pylorus or duodenal bulb stenosis on imaging. Laboratory findings showed metabolic alkalosis and hypokalemia. When the gastroscope reached the antrum, the endoscopic doctor found a disc-shaped brown object (Flammulina mushroom) in the posterior wall of the posterior antrum.

**Interventions:**

A snare was used to remove the Flammulina mushroom through the endoscopy.

**Outcomes:**

The patient’s gastric emptying completely resolved, and no vomiting symptoms recurred after 1 month of follow-up.

**Lessons:**

For young men with a history of digestive disease, when gastric retention occurs, doctors are more inclined to diagnose the obstruction caused by the ulcer based on their subjective consciousness. This is a case that did not follow this expectation. Some food without sufficient chewing couldn’t be easily digested by gastric acid.

## 1. Introduction

The most common cause of pyloric obstruction is pyloric or duodenal ulcers. Repeated ulceration leads to repeated scarring of the pylorus or duodenum, which eventually leads to deformation and narrowing of the lumen. Symptoms often manifest as nausea, vomiting, or other upper gastrointestinal obstruction symptoms. For young men with a history of digestive disease, when gastric retention occurs, doctors are more inclined to diagnose the obstruction caused by the ulcer based on their subjective consciousness. This is a case that did not follow this expectation. Rather, the final diagnosis was a chronic pyloric obstruction resulting from foreign matter. The obstruction was relieved after the endoscopic removal of the foreign matter.

## 2. Case report

**Figure 1.**
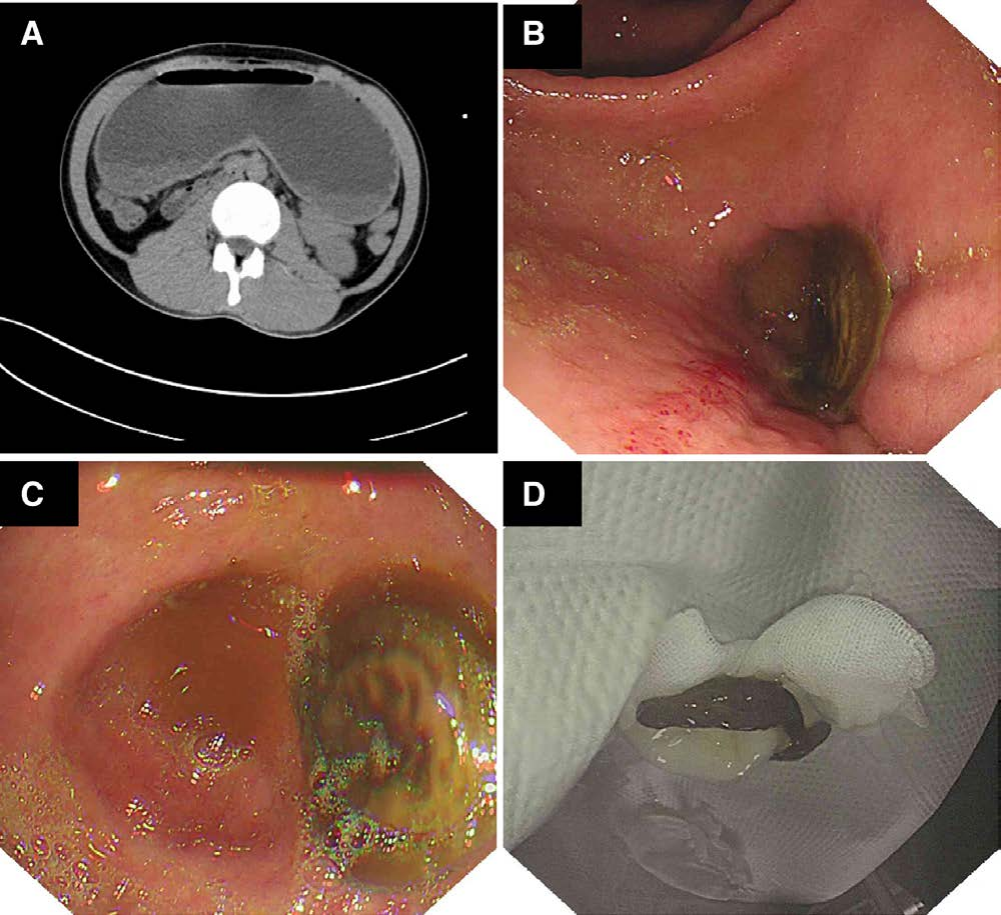
(A) The emergency CT images of the patient. (B and C) The gastroscope images of the posterior wall of the patient’s posterior antrum. (D) The in vitro observation of the pyloric obstruction.

A man in his early 30 presented to the emergency department of Shenzhen Second People’s Hospital due to recurrent vomiting for 4 years that had aggravated for 2 days. Emergency CT indicated enlargement of the gastric cavity, numerous gastric contents in the gastric cavity, and suspicion of pylorus or duodenal bulb stenosis on imaging (Fig. 1A). Laboratory findings showed metabolic alkalosis and hypokalemia. The patient’s past history was that the patient had been diagnosed with duodenal ulcer due to upper gastrointestinal bleeding 5 years prior, and he had taken regular medication for 6 weeks. After that, vomiting occurred repeatedly once every 1 to 2 months. After taking PPIs, the symptoms improved slightly, but no gastroscopy has been performed in the past 4 years to determine its severity. The patient exhibited evidence of metabolic alkalosis (pH 7.545), hypokalemia (3.28 mmol/L), and hypochloremia (81 mmol/L).

According to the patient’s current medical history, previous history and imaging examination results, the receiving doctor initially suspected that the patient had duodenal bulb deformation and stenosis caused by repeated attacks of duodenal bulb ulcers. To identify the cause of the symptoms as soon as possible, the emergency department treated the patient with potassium and chloride supplementation, intravenous nutrition and gastrointestinal decompression; additionally, emergency gastroscopy was performed immediately after the gastrointestinal decompression tube was completely drained. When the gastroscope reached the antrum, the endoscopic doctor could not find the pylorus, and a disc-shaped brown object could be observed in the posterior wall of the posterior antrum (Fig. 1B). Close-up observation was performed, as was the case for the mushroom head (Fig. 1C). A biopsy clamp was used to push the object to good mobility. Therefore, a snare was used to remove the foreign matter from the body. In vitro observation of the substance revealed that the fungus appeared to be the Flammulina mushroom (Fig. 1D). Entering the gastric cavity again, the opening and closing of the pylorus were observed, and no deformation was found in the duodenal bulb. Eventually, the pyloric obstruction resolved. After the operation, the patient had no more nausea or vomiting, and his diet returned to normal. The patient’s electrolyte levels and blood gas analysis were checked the day after the gastroscopy, and both returned to normal. We have followed up with the patient by telephone, and he has had no further gastrointestinal symptoms to date.

The reporting of this study conforms to CARE guidelines. In addition, we obtained board approval for publication. The reporting of this study conforms to the CARE guidelines (for case reports).

## 3. Discussion

The most common cause of pyloric obstruction is peptic ulcer disease.^[1,2]^ Repeated ulcer formation leads to repeated scar formation on the pylorus, which eventually leads to deformation of the pylorus, preventing food passage and causing obstruction symptoms.^[3]^ In young patients with a previous history of ulceration, gastric retention occurs. Physicians often prioritize the diagnosis of obstruction caused by peptic ulcers. This patient was no exception, and gastric retention caused by pyloric deformation and stenosis was considered at the first diagnosis. However, the final result was unexpected.

After the medical history was collected, the patient told the doctor that he had eaten Flammulina mushrooms when he had eaten hot pot with his friend 4 years prior, but he swallowed the Flammulina mushrooms without sufficient chewing. Flammulina mushrooms are also called “see-you-tomorrow mushrooms” because they cannot be digested or decomposed by gastric acid, which can also explain why the patient remains in the stomach after 4 years of intermittent vomiting.

By gastroscopy, we found the mushroom head in the stomach cavity and the stalk in the duodenum, similar to the piston, can move as the gastrointestinal tract. When the mushroom head does not completely cover the pylorus, food and gastric juice can spread slowly through the pylorus. In contrast, when the mushroom head completely covered the pylorus, pyloric obstruction is occurred, and gastric juice and food could not through the pyloric region. This can explain why the patient had intermittent vomiting and why the mushroom head completely covered the pylorus. This is related to whether the mushroom head completely covers the pylorus.

During the 4 years of coexistence of the Flammulina mushroom and the gastric cavity, gastric emptying was affected. To adapt to this change, the gastric cavity was gradually expanded, which was reflected in the abdominal CT examination. As this is a long process, the patient gradually adapts to the discomfort caused by dilatation of the stomach. The patient was admitted to the emergency department because of severe alkalosis and convulsions in his limbs, which were related to the massive loss of gastric fluid. The limitation of this study is that it is a rare case and cannot fully represent similar patient situations. Therefore, it is still necessary to determine an individual treatment plan for similar patients.

This case is the first reported worldwide case of chronic pyloric obstruction due to blockage of Flammulina mushrooms. Our team confirmed the diagnosis through endoscopy. After removal of the foreign body, the patient’s gastric emptying completely resolved, and no vomiting symptoms recurred after 1 month of follow-up.

## Author contributions

**Conceptualization:** Ruinuan Wu.

**Data curation:** Zhaohui Liu.

**Formal analysis:** Ruinuan Wu.

**Funding acquisition:** Dayong Sun, Xiangyu Wang.

**Investigation:** Zepeng Wang.

**Methodology:** Zepeng Wang.

**Validation:** Zepeng Wang.

**Writing – original draft:** Zhaohui Liu.

**Writing – review & editing:** Dayong Sun, Xiangyu Wang.
